# Too long to wait: South Asian migrants’ experiences of accessing health care in Australia

**DOI:** 10.1186/s12889-021-12132-6

**Published:** 2021-11-17

**Authors:** Manju Adhikari, Sabitra Kaphle, Yamuna Dhakal, Sabina Duwadi, Rajan Subedi, Sonu Shakya, Sunil Tamang, Mukesh Khadka

**Affiliations:** 1 LA GRANDEE International College, Pokhara Metropolitan City, Province 4 Nepal; 2 Central Queensland University, Melbourne, VIC Australia

**Keywords:** Migrant patient experience, Access to health care, Health care barriers, South Asian migrants, Culturally competent care

## Abstract

**Background:**

Migrants settling in a new country experience multiple complexities in navigating health care systems and adapting to a new way of life in the host country. In South Asia, migrating to another country for better life opportunities has been an ongoing trend and migration to Australia has significantly increased in recent years. Lower utilisation of health services and higher risks of chronic diseases among South Asian migrants poses a continuing challenge for the Australian health care system and little is known about why this demographic group does not access health services at the same rate. This study aimed to explore factors influencing access to health care by South Asian migrants in Australia.

**Methods:**

Using a mixed-method design, we conducted 62 online survey and 14 in-depth interviews with participants from four South Asian countries: Nepal, India, Bhutan, and Sri Lanka. Participants were recruited using a purposive snowball sampling approach following a standard ethical approval process. Survey data were analysed descriptively in SPSS and interview data were recorded, transcribed, and analysed thematically.

**Results:**

South Asian migrants experienced various complexities while accessing health services in Australia. The findings of this study highlighted a number of negative factors influencing their experiences of accessing health care: long waiting times for public health care, the expense of private health care, and communication problems due to socio-cultural differences. South Asian migrants also expressed their concern for a greater investment of resources into public health care to enable them to access quality and affordable care in these settings.

**Conclusions:**

Given limited evidence available to help understand factors leading to the lower utilisation of health care and higher risks of chronic diseases among South Asian migrants, this study plays an important role in highlighting social, cultural, financial, and institutional factors that are critical to designing appropriate health-care strategies. This study recommends incorporating a collaborative and culturally competent model of care to increase access to health care and thereby help reduce existing disparities in health outcomes among South Asian migrant populations.

**Supplementary Information:**

The online version contains supplementary material available at 10.1186/s12889-021-12132-6.

## Background

Disparities in health outcomes among migrants are a growing concern in developed countries including Australia, Canada, USA, and New Zealand where most migration from other countries occurs for various reasons - study, employment, safety and better life experiences [1–4]. Lower access to health care is a major challenge in addressing disparities in health outcomes globally [1, 3, 5, 6]. Australia reported lower utilisation of services and higher risks of chronic diseases among migrant populations [7–10]. A similar trend is reported in Canada [2, 3], USA [3], and New Zealand [4]. What is so far known is that reasons for lower utilisation of services among migrants in Australia is related to their inability to navigate available health services due to a range of factors [11–13].

The Australian population represents diverse cultures, ethnicities, languages, and nationalities, with people coming from over 200 countries through migration and humanitarian programs, and 29.7% of the population is overseas born [14]. According to the Australian Government, the South Asian population is categorised under the CALD (culturally and linguistically diverse) community category [1]. In the last 5 years, the South Asian population has increased rapidly in Australia comprising over 14.2% of the total overseas-born population as reported by census [15]. The South Asia region represents countries under the South Asian Association of Regional Cooperation (SAARC): Afghanistan, Bangladesh, Bhutan, India, the Maldives, Pakistan, Nepal, and Sri Lanka [16]. While most South Asians come as migrants to seek better life opportunities in Australia, their settlement experiences are impacted by multiple factors such as language differences, cultural backlash, lack of suitable employment, social isolation and experiences of different forms of discrimination [7, 17, 18].

Health is a socially constructed concept and the way that people view health impacts their decisions around seeking care and use of available health care [8, 19–21]. On one hand, there have been growing concerns around ensuring access to culturally safe health services to meet the needs of diverse population groups [8, 17, 22, 23]. On the other hand, migrant populations are struggling to navigate health systems to access information and services [24, 25]. Consequently, increasing access to health care for migrant populations to improve utilisation of care has been a continuing challenge for the health system [9, 10, 25–27].

Despite targeted programs designed to address linguistic, cultural, financial, and social barriers experienced by cross-cultural communities while accessing health care, progress towards enhancing health outcomes has been insignificant [13, 28]. Evidence suggests that Asian migrants have reported high levels of anxiety and confusion in understanding how health services operate in Australia and this has discouraged access to health care in general [13, 17]. It is encouraging that there has been ongoing advocacy to take the socio-cultural context of migrant populations into account to enable access to health care [29]. Given the limited evidence available to understand health practices of South Asian migrants living in Australia [17, 30], the influence of their socio-cultural environment is critical to determining health and wellbeing outcomes [7, 17]. Arguments are made for the health care system to play a significant role in improving accessibility to health care and enhancing migrant patients’ experiences of utilising health care so existing health inequalities can be effectively addressed [19, 31–33].

Consideration of a sense of safety among migrant populations has been critical to health care delivery as migrants develop feelings of insecurity and a fear of losing independence, and have privacy and confidentiality concerns while accessing health care in new countries [20, 34]. Other reported barriers include communication difficulties, the complexity of navigating the health system, the cost of health care, cultural differences, and the different nature of health care [5, 11, 13, 20, 22, 23]. Consequently, their health care needs are often left unmet [35–38]. Some argue that these experiences of barriers could be effectively addressed by generating positive interactions between health care providers and patients in a culturally safe, socially appropriate, and respectful environment [8, 36, 38]. In addition, health care models must consider socio-cultural needs to ensure access to migrant populations [28, 37].

The health care system of Australia includes the national health insurance system Medicare, which is designed to cover the entire population with the intention of protecting individuals from high out-of-pocket costs to access general health care. The health care system also allows a choice for all individuals to obtain private health insurance which gives more flexibility in deciding the type of care or specialists the patient can choose within private and public health care settings. Of the total eligible populations, about 45% has private health insurance to access certain types of hospital care [39]. The average waiting time for elective procedure in public hospital is from 12 to 18 months where similar procedure can be done within three to 6 months with private health cover [40].

Given limited evidence available to understand factors that influence access to health care by South Asian migrants [41–46], lower utilisation of health care and relatively higher risks of chronic diseases among these population groups has been a consistent challenge. To gain a deeper understanding of factors influencing access to health care among migrant populations, this paper focuses on the experiences of accessing health care in general among South Asian migrants in Australia.

## Methods

### Study design

This study used a mixed-method approach to generate in-depth insights from participants [47, 48]. While most of the data collected was qualitative, the quantitative data gathered through online survey provided demographic insights into the participants involved in the study. Demographic characteristics of participants enabled researchers to examine associations with the experiences to understand similarities and differences. This method was suited to gaining the trust of participants in order to share their experiences with researchers [49, 50]. Data were collected using online survey and in-depth interviews.

### Ethics and consent

This study was approved by the Human Research Ethics Committee of Central Queensland University (Approval number 020–20). Participants were provided with electronic information about the research in English, explaining the voluntary nature of participation prior to consenting to start the online survey. Interview participants were provided with an electronic copy of the information sheet to arrange an interview time. All participants provided informed consent in which survey participants provided electronic written consent and interview participants provided audio recorded verbal consent following the standard ethical procedure. Because of the restrictions to conduct face-to-face interviews due to the outbreak of COVID-19, audio recorded verbal consent was recommended and approved by the ethics committee. Participants’ decisions, privacy, preferences, choice, and confidentiality of information were maintained throughout the research process.

### Participants

Participants involved in the study were aged between 21 to 58 years. All participants had migrated from four South Asian countries - Nepal, Bhutan, India, and Sri Lanka - and had been living in the Melbourne metropolitan area for at least 1 year. We recruited participants using purposive snowball sampling because of the nature of the study [51]. Due to the imposition of restrictions and stay-at-home orders during the COVID–19 pandemic, a call for participation was made via social media channels and emails: Instagram, Facebook, WhatsApp, and LinkedIn. An expression-of-interest process was linked with the online survey to allow survey participants provide further details if they were interested to attend the interviews.

### Data collection

Data collection occurred between May and August 2020. We collected 62 responses from online survey. Online survey questionnaire included both closed and open-ended questions to explore experiences of accessing health care in Australia (Supplementary file 1). The average survey completion time ranged from 10 to 20 min. In addition, we conducted 14 in-depth interviews. Interviews provided participants with an opportunity to talk about the factors that impacted their experiences and enabled researchers to gain a deeper understanding of barriers to accessing health care by these population groups. An interview guide was developed to facilitate conversation using broad questions (Supplementary file 2). Interviews were scheduled at a convenient time chosen by participants and were audio recorded, de-identified and transcribed verbatim by the researchers. Interviews were held using telephone or video call options as per the preference of participants. Both survey and interviews were conducted in English.

### Data analysis

Survey data was extracted in SPSS and analysed for statistical patterns, relationships, and distributions of various characteristics of closed responses. Content analysis was undertaken manually to extract key themes from open-ended responses. A thematic analysis approach was used for interview data following the six-step process to derive emerging themes from the narratives [52]: familiarizing with the data, generating initial codes, searching for themes, reviewing the themes, defining and naming themes and producing the report.

## Results

In this paper, findings gathered through online survey are presented under quantitative results whereas the themes emerged from the interviews and open-ended responses are presented under qualitative results.

### Quantitative results

#### Characteristics of participants

Participants involved in this study represented Nepal (30.6%), India (30.6%), Sri Lanka (25.8) and Bhutan (12.9%). Among those, 60% were male and 40% were female and their age ranged from 21 to 58 years with nearly 68% aged between 21 to 30 years. Over 93% of participants had no reported chronic conditions and the remaining 7% reported having either diabetes or high cholesterol.

**Fig. 1:**
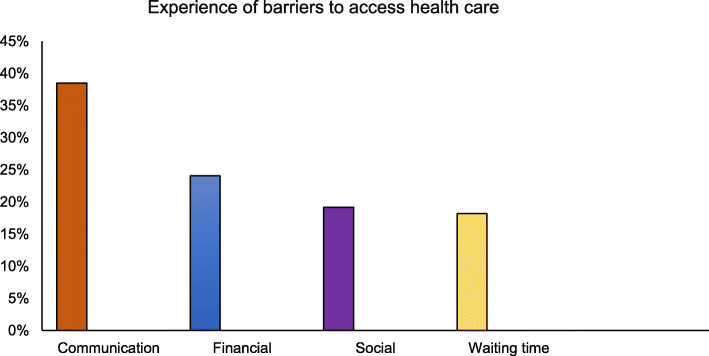
Experiences of barriers to access health care

All participants spoke their native language at home and their length of residence in Melbourne ranged from 12 months to 11 years. In terms of education, 29.0% had completed postgraduate education, 40.3% had completed a bachelor’s degree, 22.6% had completed a diploma and 3.2% had completed a certificate level of education. Most participants originally came to Australia for further education then gradually settled there. Participant experiences gathered explored multiple barriers to accessing health services by South Asian migrants in Australia. Out of 62 responses, 80% reported the experience of communication, financial, social, and waiting time barriers while accessing services in Australia (Fig. 1).

Experience of communication barrier related to language was significantly higher whereas cost of services created financial barrier to access care. Another significant result was experience of social barrier which relate to the experience of discrimination and stereotype attitude of staff in the health care setting. Long waiting time to access require care was frequently mentioned as a critical barrier to access care.

**Fig. 2:**
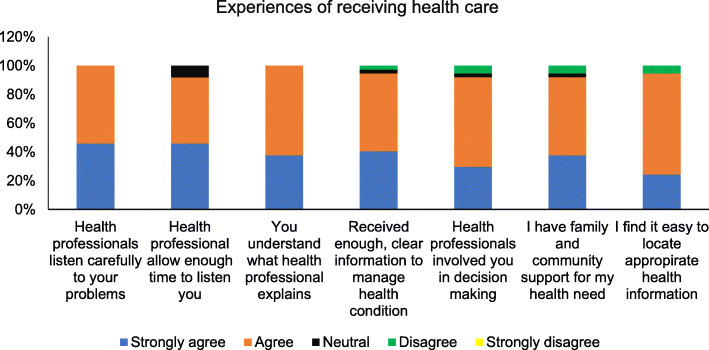
Experiences of receiving health care

Despite the experience of barriers to access health care, participants rated highly about the health professionals providing health care and the supports they have in Australia (Fig. 2).

In addition to the highly positive ratings participants provided about the use of services, access to family and community supports and ability to navigate health care information explored via survey, themes derived from the analysis of qualitative data provide further insights about the experience of barriers and satisfaction of accessing health care.

### Qualitative results

While analysing open ended responses and interview data, the following key themes emerged: too long to wait, experience of accessing public health services; expensive but reliable; experience of accessing private health services; better than home; comparative experiences of using health services; and could be done better, expectations for the future.

#### Too long to wait experience of accessing public health services

Participants shared their experiences of long waiting periods to access public services in Australia. The experience of waiting resulted either in delaying seeking services or considering private options. One participant shared her experience of attending emergency services.

“I had to go to a public hospital because of an urgent condition. After several hours of waiting, I was finally able to see the doctor. Once the doctor saw me, he told me that my problem did not require emergency treatment. They asked me to take Panadol and sent me home. I wanted treatment, that is why I went there but I was not able to get it. Instead, they gave me big bills. It was disappointing. It felt like slapping my own face. This was completely unfair. The next day, I made an appointment with one of the Bupa clinics for treatment.” Participant 1, Sri Lanka

Participants’ experiences of long waiting times became more complex when they had to pay for services. This also confirms that not being able to receive expected treatment after a long wait caused disappointment to participants. Their experiences of disappointment led to decisions to seek other services.

Another participant shared similar experiences of waiting to receive specialist appointment for treatment.

“I was referred by my GP to see a specialist. First, it took several months to get an appointment. Second, it took another few months to organise the treatment needed for my condition. It was frustrating to wait for so long. Anything could have happened during those waiting periods; the problems could have become serious. The waiting has been a difficult experience for me.” Participant 11, Bhutan

Participants found hard to manage long waiting periods, poor-quality care and expensive services. When they were not able to afford private options to minimise the waiting period, their experiences became more complicated.

One participant commented on how this scenario impacted their experience.

“I have been in a situation of about a year-long wait to get an appointment at a public hospital. Even after that long wait, I was not able to receive the treatment I needed from the public hospital. Everything was very slow. I know now why people go to private hospitals, but I cannot afford it. Public services should have a better quality of care, so people don’t need to go private.” Participant 9, Sri Lanka

On top of frustrations created by long waiting periods to receive services, these experiences raise serious questions about the reliability and quality of services delivered by public hospitals which failed to meet service expectations, and how to overcome financial barriers to care for these migrant patients.

#### Expensive but reliable: experience of accessing private health services

Some participants who were able to afford private health services to minimise delays in getting the care they needed shared their experiences. Using private health services wasn’t their first preference but long waiting periods in the public health system forced them to seek options for timely treatment of their health conditions.

“As I was not able to receive care from the public health system, I had to make an appointment with Bupa [private clinic]. It was not my first choice, but I had no other option. I was happy with my decision, as staff at Bupa explained the problem well. I was convinced by their explanations to organise necessary treatment. Their communication was good, and I felt comfortable and respected. Though I had to pay more money, I had a very good experience. This is how I like health services to treat people.” Participant 1, Sri Lanka

Bupa is a private health care provider in Australia. This participant had no prospect of receiving immediate care via the public health system, so had to make the decision to pay for private services. Another narrative highlighted the issue of high fees in order to access private health options and shared a positive experience the patient and family members had receiving treatment on time.

My sister-in-law needed surgery. The public system quoted about a 12-month waiting period, so we decided to go private. She had a stone and it might have grown bigger and become serious if we hadn’t taken the decision to take it out immediately. So, the surgery was done, and the stone was removed but we had to pay lots of money. It was hard financially as we needed to cover everything with a casual job and low income. But still it felt good to get treatment on time instead of waiting too long to be treated causing extreme stress.” Participant 7, India

For some participants, the preference for using private services was based on the promptness of care.

“If we go private, any small or big procedure can be done quickly without needing to wait. It is expensive but there are no other options or choices to make. I started going to private clinics and getting treatment on time.” Participant 9, Sri Lanka

These experiences confirm that using private services was the only option for these participants to receive timely treatment rather than a choice. They had to make significant adjustments to their financial circumstances to afford private care to manage health conditions. This raises another critical question about limited access to care for those who are economically disadvantaged and unable to overcome financial barriers.

#### Better than home: comparative experiences of using health services

Although participants raised serious complexities around managing long waiting times to receive quality of care, their experiences of using services in Australia compared favourably to their home country. This could be because most public hospitals in the South Asian region struggle to obtain resources required to offer quality health care to their communities. A few participants made such comparisons and were happy with services in Australia. One interview participant compared differences in communication among health professionals.

*“In our country [Sri Lanka], we think that doctors are like a god. Whatever they [doctors] decide, we do not question, and we just do as we are told. Our doctors there [back home] do not explain to us what they are doing. I found this does not happen here [Australia]. We can ask questions of the doctors and they explain things clearly to us. I like the services we get in Australia – they are much better.” Participant 12, Sri Lanka*

Another participant made a similar comparison about processes.

“I like the system here in Australia. They [health professionals] ask for consent before commencing any treatment, so I know what is happening and I ask for clarity. I have options to say no if I do not want to go through the procedure – this is good. Also, we do not have to pay for most services. I did not see the system working like this in our hospital back home. Our doctors there [Nepal] make decisions and carry out treatment. We have to pay for every single service we get.” Participant 4, Nepal

Better experiences in Australia were evident when participants compared the structure and way services are made available to the community.

“In India, there are different kinds of hospitals and doctors. Some hospitals known for specialist services are located in the major cities and are expensive. Same with the doctors –better doctors are based in the cities and charge more. But in Australia, I did not see that difference. All doctors are good and treat their patients equally. Regardless of whether you live in cities or rural areas, there [Australia] is better access to doctors and services.” Participant 13, India

Because of prior experiences of accessing and utilising poorly designed health care services in the home country, it is normal for these migrants to see health care provision in Australia as comparatively better. While comparisons between experiences elicited positive features of receiving health care services in Australia, participants also shared their ideas for improvement of services which could contribute to making migrant patients’ experiences more satisfying and appropriate to their social and cultural context.

#### Could be done better: expectations for future

Most health services are yet not able to meet the socio-cultural needs of communities. Participants shared expectations to address the barriers they experienced while accessing services in Australia. Most participants consistently stated their preference for more timely and affordable health services, having bilingual health professionals from the same culture, and respectful health service environments.

One participant shared their expectations for not having to wait a long time and accessing affordable services.

*“I really hope that we don’t have to wait too long to receive services. The government should provide additional resources to hospitals, so we can get treatment when needed. They should provide more nurses, more doctors, and more beds. The problems with waiting we are currently experiencing to receive care must be addressed by the government. Services should be affordable for everyone. For a developed country like Australia, they can provide better access to services.” Participant 9, Sri Lanka*

In addition to increased affordability and shorter waiting times, participants wanted access to health professionals who speak the same language and understand their cultural background.

*“I would like to see health professionals and interpreters from my own cultural background. They will understand me well if they share the same background and I can share my problems openly. This will make a big difference to my experience. Health services can match professionals with my background by asking questions when confirming appointments. They [health services] can make this work.” Participant 11, Bhutan*

Some participants experienced discrimination while seeking health care because of language and cultural differences. They clearly thought that they should not experience any form of discrimination or unequal treatment in the health service environment.

*“Health professionals [doctors, nurses] should provide clear information and make sure that we understand what they are saying. I noticed that even in the reception area, they do not provide enough information to us. I have seen them engaging in conversation with people who speak English but that does not happen to us, as we cannot speak English well. They also do not pay much attention to us. I felt discriminated against and I think this should not happen. They should respect everyone and treat others equally.” Participant 2, Nepal*

Experiences of discrimination that participants shared in this study raise a serious human rights issue that health services should seriously take into account. No one should be discriminated against in receiving care on the basis of their socio-cultural background. Health services are critical and must make a concerted effort to ensure everyone feels safe, valued, treated equally and respected.

## Discussion

This paper presented the findings of a study conducted in Australia to explore experiences of accessing health care services among migrants from South Asian backgrounds. While these migrants presented themselves as a patient to receive needed care and treatment, they experienced a range of difficulties regarding the response of the public health care system. Commonly experienced barriers included a number of factors: delays in accessing care, the high cost of care, language problems, poor quality of care, and experience of discrimination and lack of understanding of the context of migrant patients by service providers. Although service utilisation experiences of migrant patients are comparatively better in Australia, results of this study highlight gaps that health care services need to pay attention to in order to provide culturally competent care to migrant populations from South Asia.

Although health services in Australia have wider coverage and a comparatively better quality of care, the longer waits to access public services have been an ongoing issue [53, 54]. The South Asian migrants’ experiences of accessing health care services in Australia involve a complex interplay of factors resulting in mistrust of the quality of health care services that mostly originated at the level of systems. This is consistent with experiences of multiple barriers reported in other studies [9, 10, 13, 20, 54–58]. Participants shared their disappointment in long waiting periods to receive treatment, experiences of poor quality of care, financial burdens to cover the cost of health care services and discriminatory behaviour of health professionals while accessing health services in Australia.

Similar to findings reported in previous studies [59–61], this study confirms negative experiences of using public health services which has influenced decisions around accessing private health services. Even though the experience of using private services was comparatively positive, the cost of care is still a significant burden for these migrants from South Asian countries. However, compared to experiences of seeking health care services in their home countries, participants found health care services in Australia are more systematic, well designed, and suited to meet the care needs of different age groups and populations. As most South Asian migrants come to Australia from socio-economically vulnerable communities; it is not surprising that they develop positive impressions of Australian services and health care systems [41, 62, 63].

Alongside positive experiences of receiving health care, participants reported experiences of discrimination based on their language and cultural differences that resulted in mistrust of health professionals and the health care system. While previous studies reported similar consequences of discriminatory experiences [64, 65], this study revealed experiences of cultural differences in communication that raise serious questions about their capacity to be treated equally in health care settings. Other studies have suggested a need to make services responsive, culturally appropriate and respectful to migrant communities who share diverse cultural backgrounds [3, 55, 66]. Compared to mainstream population, the tendency of avoiding the use of emergency or other health care is common among the migrants and other ethnic minorities [67]. We noted similar practice of South Asian migrants which was based on their experiences of existing barriers to access health care and the nature of care they were able to receive in Australia.

Drawing on the voices of South Asian migrants settled in the metropolitan region of Melbourne, Australia, provided insights for addressing consistent financial, social, institutional, systemic, and cultural barriers to accessing quality health care services. As participants constantly described the cost of services being problematic to them, they suggested that health services should be made more affordable to everyone. Given that evidence around associations between income and the likelihood of being at risk of chronic diseases are prevalent among the South Asian migrants [68–70], it is important to make health services accessible to these population groups. Consistent with arguments made in other studies [13, 20, 54, 58, 71, 72], participants in this study strongly highlight the urgency of getting on-time care to manage health conditions and express optimism for minimising waiting times to access care.

Communication plays a critical role in ensuring positive service experiences and quality of care [11, 20, 23, 34, 58]. This study highlighted those experiences of different treatment and communication in health care settings must be effectively addressed to enable better access to care, so the community feels safe, valued, and respected when utilising available services. Having services culturally safe, appropriate, and respectful to meet the needs of communities contributes to increased service utilisation and helps address existing health inequalities among migrant populations [65, 73, 74]. Participants offered some solutions to increase service utilisation by investing more resources for service improvement, enabling access to health professionals and interpreters from the same cultural and linguistic backgrounds and creating non-judgemental and respectful service environments. These are critical components of health care [75] and can be incorporated into a culturally competent model of care where clients, families, and service providers work together to enhance the quality of experiences while receiving or providing care.

### Limitations of the study

This study provides significant insights into the experiences of South Asian migrants settling in Australia. However, the data collected in this study is limited as not all countries of South Asia are represented and the sample size is too limited to fully capture diverse perspectives. Further, all participants involved in this study speak a language other than English at home and this study was conducted in English which might have limited expression of the depth of experiences. We are not sure whether research in the first language could have influenced the results of this study. Out study did not differentiate the types or levels of services to explore the experience of barriers and satisfaction to care, so the results are limited to make specific reference to access emergency or primary care. We do understand that use of face-to-face interviews was a culturally preferred method of communication for these participants, but we were not able to do so due to the restrictions caused by the COVID-19 outbreak.

We made every effort to ensure participants felt comfortable sharing their experiences during remote interviews, but we couldn’t rule out that face-to-face interviews might have enhanced the richness of the data. For qualitative evidence, experiences shared by the participants in this study put forward a case for a better health care system which is able to identify, acknowledge, understand and provide appropriate responses to barriers in accessing health care services, but the experiences may be different to the experiences of migrants from other countries and regions who have come to settle in Australia.

## Conclusion

Most migrants experience multiple barriers while settling in a new country because of socio-cultural differences and struggle to navigate a new health system. Australia has increased migration from South Asian countries in recent years and these groups of population come with their unique cultural and social system which sometimes can be challenging. Considering the context of South Asian migrants, we explored various social, cultural, institutional, and financial factors that are influential in making decisions about utilisation of services. Consistent experiences of long waiting times, the higher cost of services, and differences in communication by service provides not only limited access to services but also discouraged service use when needed.

Although experiences of receiving services in Australia are better compared to the home country, South Asian migrants shared expectations for timely and affordable services, access to health professionals and interpreters from the same cultural and linguistic backgrounds and culturally appropriate and respectful environments across the public health system in Australia. We recommend implementation of a collaborative and culturally competent model of care which allows the involvement of patients, families, communities, and services providers to enhance positive experiences across all levels of the service delivery system. We argue that incorporating collaborative models of care with diverse perspectives helps to improve utilisation of health care services and address existing disparities in health outcomes among migrant populations.

## Supplementary Information

**Additional file 1: Supplementary file 1**: Online survey questionnaire.

**Additional file 2: Supplementary file 2**: Interview guide.

## Data Availability

The datasets used and/or analysed during the current study are available from the corresponding author on reasonable request.

## Abbreviations

USA: United Stated of America
SPSS: Statistical Package for Social Sciences
CALD: Culturally and Linguistically Diverse Community
SAARC: South Asian Association of Regional Cooperation
