# Supplementary material for: Too long to wait: South Asian migrants’ experiences of accessing health care in Australia
Source: BMC Public Health. 2021 Nov 17;21:2107. doi: 10.1186/s12889-021-12132-6 (PMC8596381; doi:10.1186/s12889-021-12132-6)
Supplement: Supplementary file 1 — Additional file 1: Supplementary file 1: Online survey questionnaire. [file 12889_2021_12132_MOESM1_ESM.pdf]

## Welcome to our survey: Information sheet

**Project Overview**
**This project is being completed by students of CQU under the supervision of Dr. Sabi Kaphle as a requirement for the Master of Public Health course. In this research, we are exploring how health knowledge is created and shared among the South Asian communities of Melbourne. We are also aiming to understand community expectations on how health services can be designed to meet their needs.**

**Participation Procedure**
**Initially, you will be invited to participate in an online survey for this study. You will also have an option to choose whether you would like to be contacted for an interview, after completing the survey. A survey that consists of a series of questions for you to answer will be made available online for you to complete. It will take approximately 20 - 30 minutes to complete the survey.**
**The interview consists of open questions about your health knowledge and your expectations on how health services could address your needs. The interview will be audio recorded with your consent. Completion of the interview will take approximately 30 - 40 minutes. The interview will be held via telephone, Zoom, WhatsApp, Skype, Viber, or messenger according to your preference.**
**Participation in this project will not affect your employment, academic standing, or association with the University.**

**Benefits and Risks**
**There is no direct benefit to you for participating in this project. However, it is expected that this project may benefit you to understand the problems and barriers you face while accessing health services in Australia and to express your expectations in order to meet your socio-cultural needs to live a healthy and happy life.**
**The research team does not envisage any risks to participants other than an inconvenience for the time spent in the interview, however, given the current health crisis, it is possible that participants may be experiencing anxiety about their own health or health of others.**

**Confidentiality/Anonymity**
**All your responses will be kept confidential and handled anonymously without making personal references. The data will be securely stored for a minimum of 15 years in accordance with the CQUniversity policy and procedure maintaining ethical standards.**

**Outcome**
**The results of this research will be incorporated into an assessable piece by the students in the form of a dissertation or report. The publication is not the primary intention of this research, but a journal article or conference paper may arise out of this work.**

**Consent**
**Your consent to participate in this project will be obtained electronically from the SurveyMonkey after you read and click the next button of the consent page.**

**Right to Withdraw**
**Your participation is voluntary. You are free to end the interview and/or survey without penalty, by**

**informing the interviewer. If you complete the interview or survey, once data analysis has commenced, it will not be possible to withdraw your data.**

**Feedback**

**A short summary of the project's findings in plain English will be available in December 2020. To request a copy, please email the project supervisor.**

**Questions/ Further Information**

**If you have any questions about this project, please contact the Supervisor.**

**Dr Sabi Kaphle**
**Lecturer in Public Health | School of Health, Medical and Applied Sciences**
**CQUniversity Australia, Level 4, 120 Spencer Street, Melbourne 3000**
**P +61 3 9616 0586 | X 50586**
**Email - s.kaphle@cqu.edu.au**

**Please contact Central Queensland University's Research Division (Tel: 07 4923 2603; E-mail: ethics@cqu.edu.au) should there be any concerns about the nature and/or conduct of this research project.**

**This project has been approved by the CQUniversity Human Research Ethics Committee, approval number 2020-020.**

## Consent Form

**I consent to participate in this research project and agree that:**

**1. An Information Sheet has been provided to me that I have read and understood;**
**2. I have had any questions about the project answered to my satisfaction by the Information Sheet and any further verbal explanation provided from the project team;**
**3. I am aware that the interview will be audio-recorded;**
**4. I understand that my participation or non-participation in the research project will not affect my academic standing, my employment or association with CQ University;**
**5. I understand that I have the right to withdraw from the project without penalty at any time up until my data has been de-identified;**
**6. I understand the research findings will be included in the researcher's publication(s) on the project and this may include conferences and articles written for journals and other methods of dissemination stated in the Information Sheet;**
**7. I understand that to preserve anonymity and maintain confidentiality of participants that fictitious names may be used in any publication(s)**
**8. I am aware that a Plain English statement of results will be sent to me if I request a copy in the section below;**
**9. I agree that I am providing informed consent to participate in this project.**

**I wish to have a Plain English statement of results sent to me at the address I provide below.**

**Dr Sabi Kaphle**
**Lecturer in Public Health | School of Health, Medical and Applied Sciences**
**CQUniversity Australia, Level 4, 120 Spencer Street, Melbourne 3000**
**P +61 3 9616 0586 | X 50586**
**Email - s.kaphle@cqu.edu.au**

* 1. What is your age?

* 2. What is your gender?

- Female
- Male
- Non-binary
- Prefer not to say

* 3. Number of other family members in Australia

- 1
- 2
- 3
- 4
- 5 or more

* 4. What is your main occupation?

* 5. Type of occupation

- Full time
- Part time
- Casual
- Unemployed

* 6. Your Weekly Income in AUD

- no income
- AUD 500 or less
- AUD 500 to AUD 1000
- AUD 1000 to AUD 2000
- AUD 2000 to AUD 5000
- AUD 5000 or more

* 7. What is your monthly family income in AUD?

- AUD 2000 or less
- AUD 2000 to AUD 4000
- AUD 4000 to AUD 6000
- AUD 6000 to AUD 8000
- AUD 8000 or less

* 8. What is your highest level of education?

- Master Degree or higher
- Bachelor Degree
- Advanced diploma and diploma
- Certificate level
- School qualification

* 9. Which is your country of birth?

- Nepal
- Bhutan
- Srilanka
- India

* 10. How long you have been living in Australia?

- 2 yrs or less
- 3 to 5 yrs
- 6 to 10 yrs
- 11 yrs or more

* 11. What is the main language you speak at home?

- Nepali
- Hindi
- Sinhala
- Dzongha
- Other (please specify)

* 12. Do you have any long-term health conditions? (Multiple Choice)

- [ ] Diabetes
- [ ] Asthma
- [ ] Hypertension
- [ ] Cancer
- [ ] Not Applicable
- [ ] Other (please specify)

## Health Knowledge Related Questionnaire

* 13. What does health mean to you and your family?

* 14. What does your community think about health?

* 15. What do you normally do to protect from illness or sickness? (Multiple Choice)

- [ ] Physical exercise
- [ ] Eating healthy food
- [ ] Not going out in bad weather
- [ ] Sleeping well
- [ ] Other (please specify)

* 16. How do you know when you are sick or not well? What particular symptoms? (Multiple Choice)

- [ ] Fever
- [ ] Cough
- [ ] Stress/ Anxiety
- [ ] Fatigue
- [ ] Muscle tenderness
- [ ] Other (please specify)

* 17. What type of treatment do you do when you are sick? (Multiple Choice)

- [ ] Home remedies
- [ ] Herbal medicines
- [ ] General Practitioner (GP) or Doctor Visit
- [ ] Other (please specify)

* 18. Do you go to the doctor (GP) or pharmacy or chemist when you feel sick? Tell us why

* 19. Do you use any home remedies when you or your family members get ill?

- ◯ Yes
- ◯ No

* 20. Please list the home remedies you use.

* 21. Why you use home remedies? Give reason. (Multiple Choice)

- [ ] It increase my immunity.
- [ ] This practice is in my family and community.
- [ ] My parents and or relatives suggests me to use.
- [ ] It has less side effects.
- [ ] Other (please specify)

* 22. Do you like smoking and or drinking?

- Smoking only
- Drinking only
- Both of these
- None of these

* 23. Does any other family member like smoking and/or drinking?

- Smoking only
- Drinking only
- Both of these
- None of these

* 24. Is smoking and/or drinking is a part of your cultural/religious occasion?

- Smoking only
- Drinking only
- Both of these
- None of these

* 25. What do you do when you feel stressed? (Multiple Choice)

- [ ] Talk with family
- [ ] Talk with friends
- [ ] Listen music
- [ ] Play
- [ ] Roam around
- [ ] Other (please specify)

## Health Access Related Questionnaire

* 26. What is the main source of health information for you? (Multiple Choice)

- [ ] Social media
- [ ] Radio
- [ ] Television
- [ ] My GP
- [ ] Phone
- [ ] Family
- [ ] Other (please specify)

* 27. What kinds of health information are you interested in and why?

* 28. Why this information is important to you?

* 29. Can you get this information easily? Explain

* 30. How long do you have to travel to reach health care?

- ○ less than 30 minutes
- ○ 30 minutes to 1 hours
- ○ 1 hour to 2 hours
- ○ 2 hour to 3 hour
- ○ 3 hour or more

* 31. How do you get there (method of travel)? (Multiple Choice)

- [ ] Private Car
- [ ] Bus
- [ ] Train
- [ ] Walking
- [ ] Tram
- [ ] Rideshare application such as uber, ola, etc

* 32. How far do you prefer to go for your health check? (Multiple Choice)

- [ ] Nearby home
- [ ] where there is General Practitioner (GP)
- [ ] where there is specialist
- [ ] where there is specialist from similar background
- [ ] Other (please specify)

* 33. Do you like visiting GP or specialists nearby when you are sick?

- ( ) Yes
- ( ) No
- ( ) Doesn't matter to me

* 34. If no, what are some reasons?

- [ ] I don't like service from nearby GP/specialist
- [ ] GP/specialist nearby aren't from similar background
- [ ] It is expensive
- [ ] Insurance doesn't cover services nearby my home
- [ ] Other (please specify)

* 35. Do you prefer to visit GP/specialist of the same gender and community background?

- ( ) Yes
- ( ) No
- ( ) Doesn't matter to me

* 36. If yes, why you prefer GP from the same gender and community background? (Multiple Choice)

- [ ] They give enough time to listen to my problem
- [ ] They understand what I mean to say
- [ ] They give me idea of accessing and using health information
- [ ] I understand what they suggest me
- [ ] I have trust on them
- [ ] They suggest me the option that is culturally acceptable
- [ ] They include me in making decision of health care
- [ ] Other (please specify)

* 37. How do you book or make an appointment with GP?

- [ ] Phone call
- [ ] Online
- [ ] Visit
- [ ] Other (please specify)

* 38. Do you like to make an appointment that way?

- ( ) Yes
- ( ) No

* 39. If no, why?

* 40. Do you need to wait to get an appointment?

- Yes
- No

* 41. If you need to wait, how long?

* 42. How do you pay for health care usually?

- ○ Medicare
- ○ Private Health Insurance
- ○ Out of pocket (cash)

* 43. Do you receive any health services in the last 12 months?

- ○ Yes
- ○ No

## Health Service Use Experience Related Questionnaire

* 44. If yes, whom did you see?

- Doctors or General Practitioner (GP)
- Medical specialists
- Other (please specify)

* 45. What health service did you receive? (Multiple Choice)

- General Checkup
- Ear checkup
- Eye test
- Back pain
- Physiotheraphy
- Other (please specify)

* 46. Were you happy with the services you received? (Explain)

* 47. Have you even been unable to receive health care needed for you conditions and why?

* 48. Please rate your response to these statements

| | Strongly Agree | Agree | Neither agree nor disagree | Disagree | Strongly Disagree |
|---|---|---|---|---|---|
| Health professionals (doctor, nurse, etc.) listen carefully to your problems | ○ | ○ | ○ | ○ | ○ |
| Health professionals allow enough time to listen to you | ○ | ○ | ○ | ○ | ○ |
| You understand what health professionals explain to you | ○ | ○ | ○ | ○ | ○ |
| You are happy with the way they check you | ○ | ○ | ○ | ○ | ○ |
| You received enough and clear information to manage your health condition | ○ | ○ | ○ | ○ | ○ |

* 49. Please rate your response to these statements

| | Strongly Agree | Agree | Neither Agree nor Disagree | Disagree | Strongly Disagree |
|---|---|---|---|---|---|
| Health professionals involve you in making decisions about the care you need | ○ | ○ | ○ | ○ | ○ |
| I have required family and community support for my health need | ○ | ○ | ○ | ○ | ○ |
| I find it easy to locate appropriate health information | ○ | ○ | ○ | ○ | ○ |

* 50. What are the common health conditions you think important to visit health services?

## Other Questionnaire

* 51. Do you see any other barriers to accessing health care in Melbourne? If so, write what they are.

* 52. How do you think these barriers could be addressed? Please write your opinion.

* 53. Is there anything else you would like to mention? Please write.

* 54. Do you like to take part in the interview?

○ Yes

○ No

○ May be
