# Supplementary material for: Too long to wait: South Asian migrants’ experiences of accessing health care in Australia
Source: BMC Public Health. 2021 Nov 17;21:2107. doi: 10.1186/s12889-021-12132-6 (PMC8596381; doi:10.1186/s12889-021-12132-6)
Supplement: Supplementary file 2 — Additional file 2: Supplementary file 2: Interview guide. [file 12889_2021_12132_MOESM2_ESM.docx]

# Project Title

# Social Construct of Health and Community Insights for Appropriate Service Model

**Interview Guide**

**Background**

This project aims to explore health knowledge and health care expectations of people from South Asian background that meet your needs There are two aspects of this research. The first aspect is to explore how health knowledge is created or shaped and shared among South Asian communities of Melbourne. Another aspect is to gather community insights on how health services could be offered to meet their needs. To get in-depth information on these aspects, we encourage the participants to do most of the talking like in the normal conversation. This enables the participants to share their experiences on the issues. Participants will be guided to talk in such a way that they answer the questions in a natural conversational form without the feeling of being interviewed. The table below contains the leading questions and the possible prompts for that question to follow during interview.

**Code: ……………**

| **Leading themes/questions** | **Possible prompts** |
| --- | --- |
| Opening | Thank you for agreeing to talk to me today. As you know, this is part of a research project about the social construct of health among South Asian communities and their insight for health service model. We are interested to explore health knowledge of your country and know your expectations from the health services that meet your needs. We hope that you will help us to understand these issues and share your experiences. This will help us to learn your experiences and the conversation will be used in our research project.  I would like to ask you some questions and you can answer them any way you like to answer. If you don’t want to answer any question then you can just say ‘I don’t want to talk about this’. I will record our conversation if you are happy for me to do so and make some notes but what we talked about today will not be known to anyone. Your name will not be used.  If you are ready for this, I would like to record our conversation. And if you like to stop the conversation at any point of time or do not want to take part in this study, you can tell me and withdraw at any time.  May I record our conversation now? |
| Introduction and background information | 1. What is your name? 2. Where do you come from? 3. How long have you been in Australia? 4. Do you have family here? 5. What do you do? Where do you work? 6. Do you know other people from your country? |
| Health knowledge | 1. What does health mean to you and why? 2. Where and how do you get health information from? 3. Do you value traditional knowledge of health? Why? 4. Do you believe on traditional healers? Why? 5. What does health and illness mean in your community context? 6. Is the health belief you and your family have like Australia? 7. What do you think people can do to remain healthy? 8. What is your opinion about smoking and drinking? 9. What is your opinion about eating healthy foods, doing physical exercise and living a happy life? 10. Do you follow food belief and cultural practices to feel better from illness? |
| Health care practice | 1. Where did you usually go when you were sick in your country? Why? 2. Where do you go when you are sick now? Why? 3. Has your health care practice changed when you come here? 4. Why did you change your health care practice? 5. What do you normally do when you feel sick? 6. Do you like going to health services to receive care here? 7. What is your experience of receiving health service here? Are you happy with the way you were treated? 8. If you were not happy with the services, could you explain why? |
| Health care needs | 1. How do know when you are not feeling well? 2. Do you have easy access to health care? If not, explain why. 3. Do you feel comfortable talking to health care providers about your problems and care you might need? If not, explain why. 4. Do you understand the information provided to you? If not, do you feel comfortable to ask for clarification? 5. Can you tell us about your experience of receiving care recently and how was it? |
| Health care expectations | 1. Are you satisfied with health services delivery system of Australia? If yes, how? If no, why? 2. What differences did you find between the health care system of your country and Australia? 3. Do you think the Australian health system is designed in such a way that meet the needs of people from your background? 4. Are you happy with the health service system available here? 5. Do you have different expectation than what you have been receiving so far? Could you tell us about it?   Discuss things with healthcare providers until you understand all you  need to  Discuss things with healthcare providers until you understand all you  need to  Discuss things with healthcare providers until you understand all you  need to  Discuss things with healthcare providers until you understand all you  need to  Discuss things with healthcare providers until you understand all you  need to |
| Barriers to seek health care | 1. Are there any issues preventing you from accessing the health care you and your family need in Melbourne? 2. Do you think the cost of health care is affordable for family from your background? 3. Do you feel that health professional understand everything what you explain about your health condition? 4. Do you understand everything what they tell you about your condition and the treatment you need? 5. Have you ever felt any form of discrimination while going to the clinic? |
| Suggestions for services | 1. What do you think the Australian health services can do to address the needs of your family and communities? 2. How do you think the services could be made more affordable, accessible and respectful to the people from South Asian communities? |
| Anything else you would like to say | Is there anything else you would like to say that e did not cover in our conversation today. |
